# Epigenetic Changes in Endothelial Progenitors as a Possible Cellular Basis for Glycemic Memory in Diabetic Vascular Complications

**DOI:** 10.1155/2015/436879

**Published:** 2015-05-28

**Authors:** Poojitha Rajasekar, Christina L. O'Neill, Lydia Eeles, Alan W. Stitt, Reinhold J. Medina

**Affiliations:** Centre for Experimental Medicine, School of Medicine, Dentistry, and Biomedical Science, Queen's University Belfast, Belfast BT12 6BA, UK

## Abstract

The vascular complications of diabetes significantly impact the quality of life and mortality in diabetic patients. Extensive evidence from various human clinical trials has clearly established that a period of poor glycemic control early in the disease process carries negative consequences, such as an increase in the development and progression of vascular complications that becomes evident many years later. Importantly, intensive glycemic control established later in the disease process cannot reverse or slow down the onset or progression of diabetic vasculopathy. This has been named the glycemic memory phenomenon. Scientists have successfully modelled glycemic memory using various* in vitro* and* in vivo* systems. This review emphasizes that oxidative stress and accumulation of advanced glycation end products are key factors driving glycemic memory in endothelial cells. Furthermore, various epigenetic marks have been proposed to closely associate with vascular glycemic memory. In addition, we comment on the importance of endothelial progenitors and their role as endogenous vasoreparative cells that are negatively impacted by the diabetic milieu and may constitute a “carrier” of glycemic memory. Considering the potential of endothelial progenitor-based cytotherapies, future studies on their glycemic memory are warranted to develop epigenetics-based therapeutics targeting diabetic vascular complications.

## 1. Introduction

The concept of glycemic memory refers to the inexorable progression of diabetic vascular complications which is linked to uncontrolled glycemia early in the disease despite a significant follow-on period of improved glycemic control. It also relates to the association between tight glycemic control early in the course of disease and prevention of progression to late macrovascular, retinopathic, and neuropathic complications, independently of future glycemic control strategies. The glycemic memory phenomenon was initially found by Engerman and Kern in diabetic dogs in 1987 [1]. This investigation was designed following preliminary clinical studies reporting that diabetic retinopathy progression could not be arrested but worsened with tighter glycemic control [2, 3] and established that diabetic retinopathy could be prevented in diabetic dogs only if tight glycemic control began within 2 months of diabetes onset. Tight glycemic control starting 2.5 years after diabetes onset did not halt diabetic retinopathy progression, and interestingly retinopathy became evident during the tight glycemic period [1]. This preclinical study was clinically validated with results from the Diabetes Control and Complications Trial (DCCT) [4] and its follow-up the Epidemiology of Diabetes Interventions and Complications (EDIC) trial [5]. The DCCT/EDIC human clinical trials have demonstrated that the level of glycemic control early in the disease process will dictate the speed of progression for diabetic retinopathy [6].

**Figure 1:**
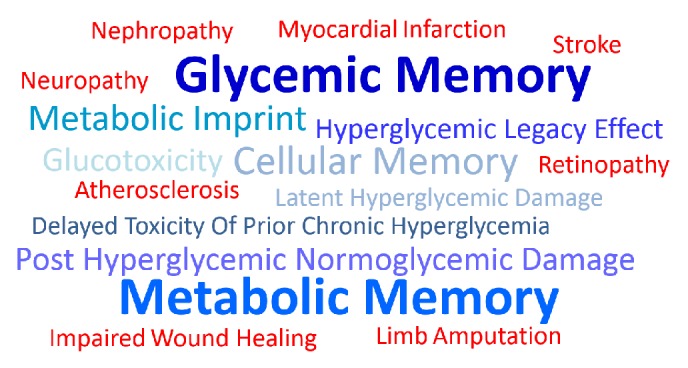
Word cloud illustrating diverse nomenclature for glycemic memory and associated diabetic complications.

Glycemic memory is often described using diverse nomenclature such as metabolic memory [7, 8], delayed toxicity of prior chronic hyperglycemia [9], metabolic imprinting [10], latent hyperglycemic damage [11], hyperglycemic legacy effect [12], and posthyperglycemic normoglycemic damage [9] (Figure 1). Metabolic memory is the broader term which refers to the delayed adverse effects triggered by prior exposure to glucotoxicity, lipotoxicity, and other metabolic imbalances. Because hyperglycemia is considered a key mechanistic driver for diabetic vascular complications and most* in vitro* experimental models have been based solely on high glucose exposure, here we endorse the term glycemic memory. However, metabolic memory is often used as an interchangeable term to describe this phenomenon, because it has been proposed that more than just tight glucose control is needed to prevent diabetic complications [13]. This review summarizes past and recent research on the role of glycemic memory in the prognosis of diabetic micro and macro vascular complications, covering from experimental* in vitro* models to human clinical trials. In addition, it discusses the relevance of epigenetics, with particular focus on how this might impact on endothelial progenitors as one of the cellular substrates of glycemic memory.

## 2. Diabetic Vascular Complications and the Glycemic Memory Phenomenon

Diabetes is an epidemic of increasing global concern as it causes serious health outcomes leading to reduced quality of life and a decreased life expectancy. Currently 382 million people (8.2% of adults) have been diagnosed with diabetes and 5.1 million diabetes-related deaths have been reported in 2013 [14]. The worldwide prevalence of diabetes has been estimated to increase up to 55% by 2035 [15]. Furthermore, due to the constant increase in the aging population, improvements in the health care system, and increased life expectancy for diabetic patients, we are likely to witness a rapid increase in the incidence of complications such as micro- and macrovasculopathy. The key cause of mortality and morbidity due to diabetes has been attributed to its impact on organs leading to outcomes such as blindness, renal failure, limb amputations, stroke, and myocardial infarction. At the heart of these vascular complications is a so-called “endotheliopathy” which is a progressive endothelial dysfunction leading to micro- and macrovascular damage [16]. Data indicating that vascular injury is responsible for diabetic retinopathy, nephropathy, and neuropathy underscore the endothelium as a cellular target susceptible to injury during diabetes.

Hyperglycemia is a clinical hallmark of both type 1 and type 2 diabetes which can be regulated by drugs that increase insulin secretion, suppress glucose release from the liver, delay glucose absorption, or increase the utilization of glucose by fat and skeletal muscle [17]. In spite of improved glucose monitoring technologies and advances in effective hyperglycemia control measures, the development of one or more vascular complications is a prospect for most diabetic patients [18, 19]. Macrovascular complications result in an increased risk of cardiovascular diseases like myocardial infarction and stroke. It has been reported that patients with type 1 and type 2 diabetes possess a two- to fourfold higher risk of vascular complications when compared to healthy individuals [20].

Glycemic memory is a concept that has gained importance and clinical relevance due to a persistent rise in diabetic complications. Here we describe some of the pioneer studies and recent research that provides evidence for the existence of this phenomenon in endothelial cells.

### 2.1. *In Vitro* Cellular Models

To study the poor reversibility of diabetic vascular complications, an* in vitro* model was designed 25 years ago using human endothelial cells that were cultured under 5 mM or 50 mM glucose for up to 3 weeks [21]. High glucose treatment induced a significant increase in the gene expression of fibronectin and collagen IV. The finding that this overexpression of extracellular matrix genes persisted even after cells were switched back to normal glucose levels constituted key evidence to establish the glycemic memory phenomenon at the endothelial cellular level [21].

A seminal dissection of the glycemic memory phenomenon came from the Brownlee lab. Transient exposure of aortic endothelial cells to high glucose levels of 30 mM for 16 hours led to long-lasting activation of the NF*κ*B p65 gene. This increase in gene expression persisted for up to 6 days after return to normal glucose levels [22]. In addition, the upregulated NF*κ*B p65 transcript also induced persistent high expression of inflammatory proteins MCP-1 and VCAM1 even when high glucose was not present [22].

Another model to study glycemic memory used human aortic endothelial cells and exposed them for 6 days to 5 mM or 25 mM of glucose as control and high glucose groups, respectively. The memory group consisted of cells treated with high glucose for the first 3 days and then normal glucose for the last 3 days. Assessment of superoxide production and peroxynitrite levels indicated that both remained significantly elevated in the high glucose and memory groups, when compared to the control group [23]. This increase in reactive oxygen species (ROS) correlated with the overexpression of p66 (Shc) which was suggested as a key driver for hyperglycemic memory in the vasculature [23].

Long-duration* in vitro* models have also been used. Human umbilical vein endothelial cells (HUVECs) were cultured for 21 days under normal glucose (5 mM) or high glucose (30 mM) [24]. Treatment for the memory experimental group in this case consisted of high glucose for 14 days followed by 7 days under normal glucose. The high glucose-treated cells showed significant increases in ROS, 8OHdG, and caspase-3. These same effects were also found in the memory group confirming that there are gene expression and functional changes at the cellular level that persist even after return to normal glucose conditions [24]. Another investigation with similar experimental design (long-term 3-week treatments) exposed retinal capillary endothelial cells to high glucose for one week and then switched them back to normal glucose for the remaining 2 weeks as their memory group. This study reported persistent increased protein expression of NF*κ*B, Bax, and PAR in the memory group that was comparable to cells treated continuously under high glucose [25]. A one-week culture under high glucose conditions was sufficient to imprint a cellular glycemic memory in retinal endothelial cells that remained unchanged for the next two weeks. Sirtuin 1 was suggested as a main modulator establishing the memory in this model [25].

It is important to highlight that most published* in vitro* models have used endothelial cells derived from a variety of vascular beds and that the initial high glucose exposures for the memory groups ranged from 16 hours to 14 days. Despite these differences in experimental* in vitro* models, similar findings have demonstrated that some endothelial gene expression changes cannot be immediately reversed even after normal glucose levels were reinstituted and constitute supportive evidence for the glycemic memory phenomenon. It is also relevant to underscore that these* in vitro* studies have identified some common findings in the memory group such as overexpression of NF*κ*B and increased ROS production, which suggests that oxidative stress acts as a key molecular driver for glycemic memory.

### 2.2. Preclinical Animal Models

The idea that tissues and cells retain memory of a previous glucose environment was firstly proposed from a study on diabetic retinopathy in dogs over 25 years ago [1]. This study lasted 5 years in total and studied 4 experimental groups: nondiabetic animals, poor control diabetics, good control diabetics, and diabetic dogs that were switched from poor to good glycemic treatment. This investigation showed that diabetic dogs that were switched to good glycemic control after 2.5 years of poor glycemic control continued to develop retinopathy in a similar way to the poor glycemic control group [1]. This provided the first experimental evidence demonstrating that diabetic retinopathy progression persisted, despite later efforts in keeping a good glycemic control.

Similarly, the sucrose-fed diabetic Cohen rat model has been used to elucidate the glycemic memory phenomenon in relation to diabetic retinopathy. Offspring that developed abnormal glucose tolerance, glucosuria, and diabetic complications were included in the study. Diabetic rats were treated with islet transplantation after 6 or 12 weeks of overt diabetes. The untreated diabetic group of rats exhibited retinal pathology characterised by increased capillary endothelial proliferation, pericyte dropout, occluded capillaries, and occasional microaneurysms. Islet transplantation after 6 weeks in the diabetic rats led to a significant reduction in pericyte loss and prevented endothelial proliferation. However, transplantation of islets after 12 weeks failed to prevent or reduce retinal vessel occlusion [26]. These findings suggested that glycemic memory is established very early in the diabetic disease process and that lack of an early intervention worsens diabetic retinopathy progression.

The streptozotocin-induced diabetic rat model was also used to investigate glycemic memory. After the establishment of diabetes, diabetic rats are followed up to 12 months. Animals were divided into three experimental groups: good glycemic control (GC), poor glycemic control (PC), and the memory group with 6 months of poor control followed by 6 months of good glycemic control (PC-GC). The number of acellular capillaries was significantly higher in the PC and PC-GC than in the GC [27]. These results, quantifying acellular capillaries, are in agreement with the findings from a study of retinal endothelial cell apoptosis showing that six months of good glycemic control in the diabetic rat model did not decrease the number of TUNEL-positive cells in the PC-GC group when compared to the PC [28]. This indicated that retinal capillary cell apoptosis continues to develop in diabetic rats even after 6 months of good glycemic control. Further studies in this diabetic rat model have described that the oxidative and nitrative retinal modifications occur early in the diabetic retina and that these changes are not easily reversed even after 6 months of good glycemic control [29].

An epigenetic link between glycemic memory and persistent proinflammatory phenotype in diabetic vascular smooth muscle cells (VSMCs) has been reported in type 2 diabetic db/db mice. This atherogenic and inflammatory phenotype persisted in these diabetic VSMCs even after 8-week* in vitro* culture under normoglycemic conditions [30]. A repressive chromatin histone methylation mark, H3K9me3, was observed to decrease at the promoters of key inflammatory genes in diabetic VSMCs when compared to control group. This was in agreement with a lower protein expression of H3K9me3 methyltransferase, Suv39h1. This study highlights the role of chromatin histone modifications associated with the glycemic memory phenomenon.

### 2.3. Human Clinical Trials Comparing Conventional versus Intensive Glycemic Control Therapies

#### 2.3.1. Type 1 Diabetes (T1D)

Among the first human clinical trials to compare an “intensified conventional” treatment with regular treatment for glycemic control in relation with the development of diabetic vascular complications was the Stockholm Diabetes Intervention Study (SDIS). SDIS enrolled 96 patients with T1D and nonproliferative retinopathy who were randomized to receive regular or intensive glycemic control [31]. Clinical data, evaluated after 5-year follow-up, indicated that intensive treatment significantly delayed the development of microvascular complications of diabetes including retinopathy, nephropathy, and neuropathy, when compared to the regular treatment.

Results from the SDIS were further supported by the seminal Diabetes Control and Complications Trial (DCCT). This is the largest and most frequently cited clinical trial comparing conventional versus intensive glycemic control in T1D. The DCCT was designed as a multicentre randomized clinical trial and enrolled 1441 T1D patients that were given conventional or intensive therapy with a mean follow-up period of 6.5 years [4]. Both primary prevention and secondary intervention were final read-outs. Glycosylated hemoglobin percentages were clearly different in the DCCT patient groups with median values of 9% and 7% for the conventional and intensive treatment, respectively. Findings indicated that the intensive therapy group benefited from a significant delay in the onset and progression of retinopathy, nephropathy, and neuropathy. While mortality did not differ between the treatment groups, severe hypoglycemia incidents were three times more frequently reported in the intensive therapy group. Due to clear and consistent results at the end of the DCCT, it was decided that all patients would be offered the intensive therapy. A new follow-up study called the Epidemiology of Diabetes Interventions and Complications (EDIC) was designed. The EDIC trial recruited 1375 participants from the DCCT. A report on 8-year follow-up indicated that previous intensive treatment group still benefited from a delayed progression of diabetic nephropathy [5]. 18-year follow-up on EDIC once more demonstrated a persistent benefit for the original intensive therapy group when compared to the conventional one on retinopathy progression [32]. However, reported risk reductions over 18 years were smaller than the ones reported in earlier follow-ups. The most recent report on DCCT/EDIC at 30 years showed a durable effect of initial assigned therapies despite a loss of the glycemic separation [6]. This represents consistent reliable data recognizing the important and persistent role that glycemic memory plays in the development and progression of vascular complications in T1D patients.

Considering the multiple clinical reports on glycemic memory, in order to define optimal clinical guidelines for glycemic control in T1D patients, a systematic review was performed to compare intensive glucose control versus conventional glucose control. This report included 12 clinical trials and a total of 2230 patients [33]. The main conclusion was that tight glycemic control significantly reduces the risk of developing retinopathy, nephropathy, and neuropathy. Therefore, tight blood sugar control is recommended in young T1D patients as early as possible after diagnosis in order to reduce the risk of developing microvascular complications. However, benefits appear to become less evident if complications are already present, and there is lack of evidence to demonstrate a beneficial effect of tight glycemic control in older patients.

#### 2.3.2. Type 2 Diabetes (T2D)

Human clinical trials assessing T2D patients by interventional studies to compare conventional versus intensive glycemic control have led to conflicting results and controversy. Here we discuss some of the major outcomes of these trials.

The Kumamoto prospective clinical trial enrolled 110 Japanese patients that were randomized to receive conventional or intensive treatment [34]. Clinical evaluations took place every 6 months for 6 years. Concluding data highlighted that the intensive glycemic control delayed the onset and progression of diabetic retinopathy, nephropathy, and neuropathy. These results were supported by the Danish Steno-2 study that recruited 160 T2D patients with microalbuminuria for an interventional trial to compare conventional and intensive treatments. Mean follow-up was 7.8 years, and a significant decline in glycosylated hemoglobin was recorded in the intensive group. This study concluded that patients receiving intensive treatment had a significantly lower risk of cardiovascular disease, nephropathy, retinopathy, and autonomic neuropathy [35].

Similar results were reported in the UK Prospective Diabetes Study (UKPDS 33). UKPDS studied 3867 patients who were randomized to receive intensive treatment with a sulphonylurea or a conventional treatment with diet. Follow-up over 10 years indicated that the group with intensive treatment benefited from a significant decrease in the risk of microvascular complications. Interestingly no differences were reported for macrovascular disease [36]. A further UKPDS posttrial monitoring on 3277 patients was carried out for 5–10 years with no randomized treatment groups. Glycated hemoglobin level differences among UKPDS groups were lost within one year of the posttrial. Although glycemic levels were comparable, data indicated that there was a persistent reduction in microvascular complications in the UKPDS original group that received the intensive treatment 10 years earlier. Furthermore, although the UKPDS did not find any difference in terms of macrovascular complications, the posttrial identified an emergent risk reduction for myocardial infarction during the follow-up [37]. These results showed that clinical benefits remained for up to 10 years after the end of randomized interventions. This represents proof-of-concept for the “legacy effect” as authors named the glycemic memory phenomenon.

The controversy was raised by the Veterans Affairs Diabetes Trial (VADT) that reported no differences between standard and intensive glucose controls in terms of development of microvascular complications or major cardiovascular events. VADT studied 1791 military veterans with poorly controlled T2D for a median follow-up of 5.6 years [38]. A substudy using 858 patients from the VADT focused on retinopathy progression and found that a poor glucose control at baseline was associated with increased risk of progression of retinopathy [39]. These data suggest that glycemic memory might have played a role early in the disease pathogenesis and that the interventional VADT study was not able to significantly reverse it.

More conflicting results appeared with the Action in Diabetes and Vascular Disease: Preterax and Diamicron Modified Release Controlled Evaluation (ADVANCE) trial which enrolled 11,140 patients for five-year follow-up comparing intensive and standard glucose control [40]. ADVANCE concluded that while there was a reduction in nephropathy incidence in the intensive treatment group, there were no significant differences on retinopathy or major cardiovascular events.

The Action to Control Cardiovascular Risk in Diabetes (ACCORD) trial revealed very compelling results that lead to its early closure. 10,251 patients were randomized to receive intensive or standard therapy. After 3.5 years, clinical data indicated that the group receiving intensive therapy had a significant increase in mortality and no reduction of major cardiovascular events [41]. This study highlighted, for the first time, the potential harm of the intensive glucose treatment in type 2 diabetic patients.

The trials UKPDS, VADT, ADVANCE, and ACCORD showed important but conflicting results. Some of the differences among them might be explained by the design of the trials [42], history and severity of diabetes, presence of complications, glycemic control history prior to the trials, and target patient population. For example, a recent post hoc analysis of the VADT trial divided its patient population into major ethnic groups. This demonstrated that while intensive glycemic control reduced the risk for cardiovascular events in Hispanics, this was not the case for non-Hispanic Whites and non-Hispanic Blacks [43].

In order to gain consensus for clinical guidelines, the Global Task Force (GTF) on glycemic control has reviewed and discussed these clinical trials. The key recommendation is that the intensive blood glucose control may benefit certain patient populations with moderate diabetes duration and/or no preexisting cardiovascular disease [44]. This is in agreement with the idea that the glycemic memory phenomenon is established early in the disease development. A long-standing poorly controlled diabetic patient with many vascular complications is likely to have a stronger predisposition for future disease progression which becomes harder to reverse and treat.

Although the debate on the importance of glycemic control and the development and progression of micro- and macrovascular complications within diabetes types and phenotypic subgroups might remain unsettled [45], the broad conclusion from such clinical trials and their follow-up studies (Table 1) serve as compelling evidence that glycemic memory is a robust clinical phenomenon that impacts on the vasculopathic risk in diabetic patients.

## 3. Molecular Keepers of Glycemic Memory in Endothelial Cells

### 3.1. Mechanisms for Diabetic Angiopathy

**Figure 2:**
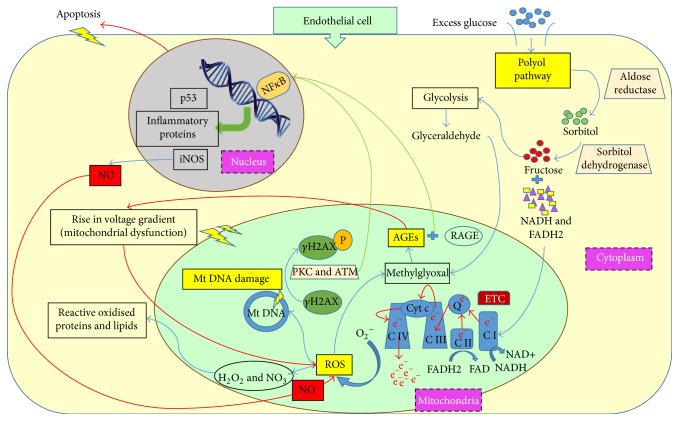
Mechanisms of hyperglycemia-induced endothelial dysfunction. Key processes responsible for hyperglycemia-induced endothelial dysfunction include the polyol pathway, reactive oxygen species (ROS) formation, and advanced glycation endproducts (AGEs) formation. The excess glucose in endothelial cells enters polyol pathway; the electron donors like reduced nicotinamide adenine dinucleotide (NADH) and Flavin adenine dinucleotide (FADH2) accumulate in the mitochondria, thus affecting the electron transport chain; the excess electrons increase ROS in mitochondria; ROS triggers accumulation of AGEs; ROS and AGEs create mitochondrial DNA damage and mitochondrial dysfunction; protein kinase C (PKC) and AGE mediated activation of nuclear factor kappa B (NF*κ*B) activate the expression of inflammation proteins, tumor suppressor p53, and inducible nitric oxide synthase (iNOS); increased nitric oxide (NO) by iNOS is highly reactive with superoxide anions; the peroxynitrite thus generated acts as a strong oxidant and completes the vicious cycle of oxidative stress by increasing ROS production; accumulation of AGEs also increases ROS production independent of glucose levels.

The vascular complications that develop in diabetic patients result from a biochemical-based injury and an impairment of protective factors [17]. The mechanisms by which hyperglycemia damages vascular cells have been studied for many years, and it is now accepted that pathogenesis is anchored in four basic mechanisms: PKC activation, increased formation of advanced glycation endproducts (AGEs), increased glucose flux through the polyol pathway, and increased hexosamine pathway activity [46, 47]. Moreover, the impairment of protective factors in diabetes such as resistance to insulin, decrease of endogenous antioxidant enzymes, and dysfunction of endothelial progenitors is as important as the injury mechanisms [17] and might provide novel therapeutic opportunities. Here, we focus our discussion on oxidative stress and AGE formation because these two molecular mechanisms (Figure 2) have been frequently associated with the glycemic memory phenomenon.

### 3.2. Oxidative Stress

Hyperglycemia leads to increased glucose levels within cells, tissues and in the extracellular space. Specifically in the endothelium, there is increased transport of glucose into the cytoplasm and an enhanced glycolytic metabolism. This escalates formation of oxygen free radicals derived from the mitochondria which can occur simultaneously with depressed activity of endogenous antioxidants which, together, contribute to increased oxidative stress [46, 48].

Free radical production is the starting point for a cascade of events that eventually lead to diabetic complications. Oxygen free radicals produced in the mitochondria of vascular cells trigger the activation of protein kinase C (PKC), increase of polyol and hexamine pathway fluxes, and production of AGEs [49, 50]. In fact, increased production of ROS, in particular superoxide, has been proposed as the unifying mechanism for diabetic complications [46, 47]. Hence, the complex interconnected pathway crosstalk that starts with free radical production has been named the “vicious cycle of metabolic memory” [13].

ROS can also derive powerful oxidants such as peroxynitrite and hydrogen peroxide, which oxidise various lipids and proteins [51], and can induce mitochondrial and DNA damage [52]. Although superoxide anions are unstable species with half-life of only a few minutes, the products resulting from their reactions with molecules like proteins, lipids, and nucleic acids persist for prolonged periods of time, thus affecting cellular functions almost permanently [13]. This was demonstrated in a diabetic rat model, where reinstitution of normoglycemia for 7 months after 2 months of hyperglycemic stress reduced the levels of retinal oxidised lipids, but not 3-nitrotyrosine (3-NT), which is a protein adduct of ROS. Interestingly, 7 months of normoglycemia after 6 months of hyperglycemia did not result in any significant reduction in retinal oxidative stress or 3-NT [29].

Hyperglycemia-induced oxidative stress in endothelial cells has been reported to upregulate plasminogen activator inhibitor 1 (PAI1) preventing fibrinolysis [53], downregulate the platelet inhibitor prostacyclin [54], and increase production of extracellular matrix [55]. These events collectively increase the adhesion of macrophages and platelets to the endothelium. Infiltration of macrophages into the endothelium increases oxygen free radical formation in mitochondria causing an additional increase of oxidative stress.

It has been demonstrated that ROS mediates the persistence of vascular stress after glucose normalization in an* in vitro* model using endothelial cells. Cells were exposed for 2 weeks to continuous high glucose and returned to normal glucose conditions for the last week. Induction of high glucose stress markers such as PKC, NADPH oxidase, Bax, 3-nitrotyrosine, and fibronectin persisted in endothelial cells even after 7 days of returning to normal glucose levels. Reduction of ROS at the mitochondrial level was an efficient approach to erase this metabolic memory [48].

### 3.3. Advanced Glycation Endproducts (AGEs)

AGEs result from the nonenzymatic irreversible reaction between reducing sugars and amines of proteins, nucleic acids, and lipids. Methylglyoxal (MGO), a dicarbonyl by-product of glycolysis, increases extensively in diabetes. MGO is highly reactive towards proteins and forms AGEs [56]. When MGO targets mitochondrial proteins, it causes mitochondrial dysfunction and excessive ROS production [57, 58]. AGEs induce nicotinamide adenine dinucleotide phosphate (NADP) reduction, thus leading to generation of more reactive superoxides [59]. Since enzymatic removal of glycation from AGEs has not been reported (except for HbA1c), it is accepted that AGE formation is an irreversible process. Therefore, AGEs will persist in diabetic tissues even with a tight glycemic control. In addition, AGEs are reported to elevate ROS production, independently of hyperglycemia [59]. Considering all this information, AGEs are likely to be one of the components of glycemic memory that leads to diabetic complications. AGEs require hyperglycemia for their formation, but once irreversibly formed their persistent presence can activate oxidative stress and inflammatory mechanisms to drive the development of vascular complications.

Accumulation of AGEs in skin collagen even after HbA1c normalization in 211 diabetic patients from DCCT/EDIC trials suggests that AGEs are reliable biomarkers of glycemic memory that correlate with the risk of diabetic retinopathy progression [60]. AGE levels have also been shown to correlate with heart failure and cardiovascular events [61]. Therefore, AGEs serve as biomarkers for the cumulative burden of tissues exposed to dyslipidemia, oxidative stress, and inflammation, in addition to hyperglycemia [62]. We believe that AGEs can be clinically useful for the management of diabetic patients as prognostic biomarkers for the development of complications, while scientifically AGEs represent reliable markers for glycemic memory.

## 4. Epigenetics to Explain Glycemic Memory

### 4.1. Epigenetic Basic Mechanisms

Epigenetic processes are described as changes in gene expression and phenotype caused by alterations in the genome that do not involve changes in DNA sequence [63]. Epigenetic mechanisms can be widely classified based on their short- or long-term effect. The short-term effect often involves a rapid response to varying environmental factors and is nonheritable in nature. The long-term effect on the other hand creates a persistent change that is stored as “memory” and passed on to the offspring, usually in response to excessive exposure to long acting stimuli [64]. However, it has been reported that even a transient change in the microenvironment can induce persisting epigenetic effects [22].

Epigenetic mechanisms are divided into three main categories: posttranslational histone modifications (PTHMs), DNA methylation, and microRNA-mediated translational control. PTHMs comprise a complex variety of biochemical reactions such as acetylation, methylation, phosphorylation, and ubiquitination. A combination of different PTHMs collectively acts in unison to create a local chromatin modification pattern. Histone acetylation of lysine residues in H3 and H4 subunits results in euchromatin conformation (active state). This reversible acetylation reaction is catalyzed by the enzyme histone acetyl transferase (HAT), while the deacetylation is induced by the enzyme histone deacetylase (HDAC) [65]. Deacetylation converts euchromatin to heterochromatin (inactive state) and consequently causes transcriptional silencing. Histone methylation is another important PTHM where the enzyme histone methyl transferase (HMT) adds methyl groups from S-adenosyl methionine to the amine terminal of lysine or arginine [66–68]. Unlike histone acetylation, histone methylation activates or silences transcription based on the position of lysine or arginine residues and the number of methyl groups added. For example, mono-, di-, and trimethylated H3K4 result in euchromatin, while mono-, di-, and trimethylated H3K9, H3K27, and H4K20 result in heterochromatin [69].

DNA methylation is characterized by the addition of a methyl group to the cytosine residue in the CpG dinucleotide sequence by DNA methyl transferase enzymes (DNMTs). The extent of DNA methylation at CpG islands (genomic regions with high density of CpG dinucleotides) located in promoter regions determines the transcription status of the gene [70]. DNMT1 classically functions as a maintenance DNMT as it conserves the methylation pattern during replication, while DNMT3A and DNMT3B create* de novo* methylation marks [71]. DNA methylation at promoter regions is usually inversely associated with regulation of gene expression. Therefore hypermethylation is associated with a blockade of normal gene expression [70]. DNA demethylation is another important phenomenon observed during embryogenesis and certain disease conditions. The demethylation process often results from absence of methylation pattern maintenance by DNMT1 (passive demethylation) or enzyme mediated excision of methylated cytosine (active demethylation). A class of enzymes called demethylases belonging to the family of MBD (methyl CpG-binding domain) proteins mediates the active demethylation process [72]. DNA methylation/demethylation is a dynamic process occurring simultaneously that regulates cellular functions and selectively triggers or represses gene expression.

It has been established that there exists a crosstalk between PTHMs and DNA methylation and that they are highly interdependent in determining gene expression status [73]. For example, low DNA methylation combined with H3K4 methylation and acetylation of H3K and H4K results in euchromatin structure. Contrarily, high DNA methylation combined with H3K9 methylation and deacetylation of H3K and H4K results in heterochromatin structure [74]. MicroRNAs (miRNAs) constitute a very different class of epigenetic controls. These single stranded noncoding RNA molecules interact with transcribed mRNA to inhibit translation, unlike PTHMs and DNA methylation, which act on the genome before transcription. miRNAs are transcribed independently and are present in the intergenic regions, exons, or protein coding introns. miRNAs bind to the 3′ untranslated region (3′UTR) of targeted mRNA and inhibit translation by either degradation of mRNA or destabilization by cleavage and deadenylation [75].

### 4.2. Specific Epigenetic Changes Associated with Glycemic Memory

#### 4.2.1. Histone Modifications

The association of certain histone modifications and glycemic memory was confirmed when a cohort of patients from the DCCT/EDIC trial follow-up were investigated. This study provided evidence of increased acetylation of H3K9 at promoters of genes related to interferon regulatory factors, inflammation, apoptosis, NF*κ*B pathway, and ROS in monocytes of 30 patients from the conventional diabetic control group. These epigenetic changes were coupled to prolonged upregulation of STAT1, TNF*α*, and IL1A and correlated clinically with occurrence of chronic diabetic complications [76].

A correlation between increased ROS and persistent histone modifications was demonstrated using rat retinal endothelial cells. Persistent reduced levels of H3K4me1 and H3K4me2 and increased recruitment of lysine specific demethylase, LSD1, were observed at the promoter of superoxide dismutase 2 (SOD2) gene in rats maintained at poor glycemic control (6 months of poor control) and the memory group (3 months of poor control and 3 months of good control). Consequent SOD2 decrease was associated with the development of diabetic retinopathy over time [77]. It has also been reported that in retinas from diabetic rats there was a significant increase of H4K20m3 at the promoter and enhancer of SOD2 which was in agreement with an upregulation of methyltransferase, SUV420h2. Most importantly, reversal of hyperglycemia failed to prevent these changes, suggesting the existence of glycemic memory in the diabetic retina driven by histone modifications [78]. Changes in the levels of histone modifying enzymes have been demonstrated in the same experimental model. Retinal endothelial cells from the poor control and memory group showed a significant increase in HDAC levels and decrease in HAT levels [79]. These changes in histone acetylation induced by hyperglycemia remained unchanged even after a 3-month return to normoglycemic conditions, thus confirming the existence of metabolic memory in retinal endothelial cells [79].

In addition, there are several reports highlighting that histone modifications represent an important mechanism triggered by hyperglycemia that leads to endothelial dysfunction (Table 2). For example, the repressive mark H3K9me3 was significantly decreased at the promoters of inflammatory genes in diabetic rat VSMCs. The resulting proinflammatory and atherosclerotic phenotype persisted even after culturing the cells in normal glucose levels for several passages [30]. Human microvascular endothelial cells exposed to high glucose exhibited increased levels of H3K4me1 and reduced levels of H3K9me2 and H3K9me3 at the promoter of NF*κ*B p65. This combination of histone methylation marks induced upregulation of NF*κ*B p65 expression which triggered the transcription of numerous inflammatory cytokines [80]. Similarly, a study on human aortic endothelial cells acutely exposed to high glucose reported that the histone methyltransferase Set7 was mobilized to the p65 promoter, resulting in a significant increase in H3K4 monomethylation [22]. Methyltransferases Set7 and SUV39h1 and demethylase LSD-1, recruited to the promoter of NF*κ*B, generate respective histone methylation changes [80]. Interestingly, the H3K4 methyltransferase Set7 has been proposed as a critical mediator of the phenomenon of glycemic memory [81]. Set7 accumulated in the nucleus of endothelial cells in response to hyperglycemia and drove activation of numerous proinflammatory genes that persisted even when switched back to normoglycemic conditions. Set7/9 have also been proposed as therapeutic targets to modulate epigenetic marks in diabetes. Knockdown of Set7/9 in monocytes was reported to significantly repress NF*κ*B and its downstream inflammatory genes like TNF*α* and interleukins. In addition, monocyte adhesion to vascular endothelial and smooth muscle cells was also prevented, thus protecting the endothelium from diabetes induced inflammation [82]. However, knockdown of Set7/9 did not reduce the H3K4 methylation at the promoters of the downstream inflammatory genes. This suggests that loss of Set7/9 is compensated by other histone methyltransferases like Set1, SMYD3, and MLLs [69].

The histone acetyltransferase cofactor, p300, has been shown to regulate the hyperglycemia-induced gene expression changes in HUVECs. Cells incubated in 25 mM glucose for 24 hours were reported to exhibit increased expression of p300 which accumulated at the promoters of endothelin-1 (ET-1), VEGF, and fibronectin along with inducing phosphorylation of H2AX and histone acetylation. Inhibition of p300 via siRNA led to decreased mRNA levels of ET-1, fibronectin, and VEGF due to reduced histone acetylation at their promoters [83]. HATs and HDACs often act in complexes with corepressors or transcription factors and exhibit high specificity for acetylation sites, thus making them complex to use as drug targets [84]. However, some Histone deacetylase inhibitors (HDIs) used in cancer have been demonstrated to inhibit all the HDACs [85]. Sirtuin 1 (SIRT1) is a class 3 HDAC that has been linked with angiogenesis [86]. This deacetylase enzyme is observed to be significantly downregulated in endothelial cells exposed to high glucose [87]. In addition, SIRT1 is also significantly downregulated in senescent endothelial progenitor cells (EPCs); and its upregulation using the SIRT1 activator resveratrol proved helpful in rescuing the EPC senescent phenotype [88].

The expression of eNOS, an essential enzyme for endothelial cell function, has been reported to be controlled by a specific histone code described as H3K9ac and H3K12ac along with H3K4me2 and H3K4me3. This histone code acts like a switch for eNOS expression and is reversed in nonendothelial cells [89].

Treatment of endothelial cells with a H3K4 methylation inhibitor methylthioadenosine resulted in decreased eNOS expression, while treating nonexpressing cells with histone deacetylase inhibitor, trichostatin-A, increased eNOS expression [89]. The H3K27me3 mark at eNOS promoter was described as a repressor for eNOS expression in early EPCs. A combination of trichostatin-A and DNMT inhibitor, 3-deazaneplanocin, was effective in removing this repressive histone code [90]. Since impairments in eNOS-related pathways are associated with endothelial dysfunction, it is possible that modulation of epigenetic marks at the eNOS promoter could become a potential target for treating endothelial dysfunction.

#### 4.2.2. DNA Methylation

DNA methylation has not been investigated in endothelial cells under diabetic conditions as extensively as histone modifications. However, there is some interesting evidence to suggest a role for DNA methylation in directing progression of diabetic vascular complications (Table 2). For example, a genome-wide DNA methylation analysis from whole blood of type 1 diabetic patients with or without renal disorders identified hypermethylation of UNC13B promoter in the diabetic nephropathy group. This is relevant because UNC13b is critical for regulation of glomerular apoptosis and has been recently implicated in the pathology of diabetic nephropathy [91]. A recent study also reported a role for DNA methylation in the development of diabetic foot ulcers. Global hypomethylation was observed in diabetic foot ulcer fibroblasts when compared to nondiabetic counterparts. Functional enrichment analysis highlighted differential methylation of gene clusters involved in angiogenesis, myofibril function, and extracellular matrix. All of these nodes are directly related to the wound healing process and comprise genes such as COL4A1, PLAU1, and FGF1. These DNA methylation changes persisted even after culturing the diabetic patient fibroblasts for several passages under normal glucose levels [92].

DNA methylation has been recently associated with glycemic memory and progression of diabetic retinopathy in a diabetic rat model. Using diabetic retinal tissue and cultured retinal endothelial cells, it was found that POLG1 promoter was hypermethylated in the diabetic group, in both retinal tissue and cultured cells. This hypermethylation continued to persist in both* in vitro* and* in vivo* experimental models of glycemic memory [93]. Furthermore, DNMT activity was significantly increased in the diabetic and memory groups. These data indicated that after a 3-month period of hyperglycemic insult there was increased DNA methylation of POLG1 promoter coupled with increased DNMT activity that persisted for further 3 months even when normal glycemic control was reinstituted. Although this evidence shows that diabetes clearly has an effect on DNA methylation, it is important to acknowledge that there are many other factors that can modulate DNA methylation profiles. For example, disturbed blood flow has been demonstrated to significantly change the DNA methylation patterns of murine arterial endothelial cells by an increased expression of DNMT1 and promoter hypermethylation of 11 proatherosclerotic genes [94].

#### 4.2.3. miRNAs

miRNAs are an important class of gene expression regulators and have been widely implicated in diabetic pathology. miRNA profiling of 80 type 2 diabetic patients compared with age and gender matched controls showed overexpression of miR-28-3p and underexpression of 12 miRNAs (miR-223, miR-320, miR-486, miR-150, miR-24, miR-21, miR-29b, miR-20b, miR-15a, miR-126, miR-191, and miR-197). These miRNA markers enabled prediction of diabetes prognosis for 70% patients [95]. A recent comprehensive review has described various groups of miRNAs that play a deterministic role in the development of diabetic complications [96].

Despite the extensive number of studies profiling miRNAs in diabetic tissue, few focus on glycemic memory. This might be due to the intrinsic short-term effects and fast kinetic mechanisms of miRNAs, while glycemic memory requires long-term irreversible mechanisms.

The use of miRNA inhibitors such as antagomirs is being investigated as a novel approach to treat several metabolic disorders. The specificity of antagomirs makes them efficient candidates to be used for targeted deletion of miRNAs without disturbing the balance of other miRNAs with similar structure, even at the single nucleotide polymorphism level [97]. However, miRNA knockdown requires multiple doses at regular intervals because antagomirs erase a specific group of miRNAs for up to only 20 days [98]. Nevertheless, future research based on using miRNA inhibitors might assist in the development of new therapies for diabetic vascular complications.

## 5. Endothelial Progenitors and Glycemic Memory

### 5.1. Endothelial Progenitor Cells

Endothelial progenitor cells (EPCs) are a subpopulation of low-frequency cells found in circulation. They are said to originate from bone marrow and navigate through the circulation following chemokine signalling to reach sites that require neovascularization and vascular repair [99]. Over the last decade, EPCs have been thoroughly examined due to their intrinsic potential to promote and contribute to new blood vessel formation. They may also be used as potential biomarkers to identify onset and progression of vascular disease [100] and to study vascular biology and pathogenesis in a Petri dish [101]. EPCs were first identified in 1997 in a seminal study by Asahara et al. who proposed that angioblasts and hematopoietic stem cells arise from a common precursor as they possess similar surface antigen profiles that include CD34, Tie2, and Flk-1 [102]. EPCs were isolated by sorting CD34^+^ cells and plating them on fibronectin-coated Petri dishes. The cells that emerged were found to express endothelial markers CD31, VEGFR2, Tie2, and E-selectin and when tested in an ischemic limb model they were found to incorporate into sites of active angiogenesis [102].

#### 5.1.1. Controversy around Different Subsets of EPC

Since Asahara et al.'s discovery [102], over a decade ago, there has been a considerable amount of controversy surrounding the EPC field due to the results of preclinical studies being largely inconsistent when compared to clinical trials. This is most likely due to the fact that heterogeneous and different populations of cells classed as EPCs have been used in experimental models and trials. The problem stems from the fact that there is no definitive cell surface marker used to identify EPCs that clearly distinguishes them from hematopoietic cells and mature vessel wall endothelial cells [103].

Two main approaches have been used to isolate EPCs. The first is (A) cell surface selection using progenitor and endothelial markers. The main markers used for EPC selection are progenitor markers such as CD34 and CD133 in combination with an endothelial marker such as VEGFR2. The second is (B) differential cell culture methodologies. It is now well accepted that using such* in vitro* isolation methods leads to the identification of at least two main and distinct subsets of cell that exhibit very different morphological and angiogenic properties [104, 105]. The first cell type arises within one week of culture as spindle-shaped cells. They uptake acetylated LDL, bind lectin, and express endothelial markers CD31 and VEGFR2. These are commonly known as early EPCs (eEPCs) due to their early appearance in culture. eEPCs are often termed proangiogenic monocytes/hematopoietic cells, myeloid angiogenic cells (MACs) [104, 106], and circulating angiogenic cells (CACs). The second subset of cells arises much later in culture, at around 2–4 weeks, and exhibits cobblestone morphology. They display unambiguous commitment to endothelial lineage and are often referred to as endothelial colony forming cells (ECFCs) [107, 108] or outgrowth endothelial cells (OECs) [109]. In spite of falling under the term EPCs, the two cell subtypes, eEPCs and ECFCs, have been shown to possess very different molecular profiles and functional characteristics [105]. Evidence in the literature suggests that only ECFCs represent the* bona fide* EPC owing to their unequivocal expression of a wide range of endothelial markers (thrombomodulin, CD146, CD34, VE-cadherin, CD31, and vWF), negativity to hematopoietic markers (CD45 and CD14), endothelial functionality, and significant proliferative potential [109, 110]. ECFC-like cells can also be generated from induced pluripotent stem cells (iPSCs) and human embryonic stem cells (hESCs). Pluripotent stem cell-derived ECFC-like cells displayed similar phenotypic and functional characteristics to umbilical cord blood-derived ECFCs. iPSC-derived ECFCs maintained an endothelial phenotype, displayed high clonal proliferative potential, and importantly formed vascular structures in multiple animal models of ischemia [111].

#### 5.1.2. Sources for Isolation, Mobilization, and Niches

EPCs can be isolated from adult peripheral blood, bone marrow, and umbilical cord blood. While umbilical cord blood remains the classical source for isolation of ECFCs, other tissues such as placenta, lungs, and white adipose tissue have also been reported as alternative sources [112–114]. The mobilization of EPCs from bone marrow to sites of vascular trauma is mediated by metalloproteinases that release the precursors from the stroma followed by upregulation of adhesion molecules and factors like vascular endothelial growth factor (VEGF) and granulocyte colony stimulating factor (G-CSF) that assist trafficking of the cells through circulation and finally lead to subsequent homing [115]. EPCs are also believed to be present in additional niches like the vascular wall [116]. A specific “vasculogenic zone” has been reported to be present in all adult blood vessels that might act as a source of EPCs [117, 118].

#### 5.1.3. Therapeutic Potential

EPCs may be used for therapeutic purposes to aid regeneration of ischemic tissues due to their ability to migrate to remote areas and promote new blood vessel formation in areas that have suffered vascular insufficiency [119]. There are various advantages in using these cells as they can be isolated from the patient and expanded* in vitro* to be delivered locally at the site of injury as autologous cells that avoid immunogenic responses. Under hypoxic conditions, EPCs have the ability to mobilize from their vascular niche into the circulation where they can target and home to specific ischemic tissues. HIF-1*α* and SDF/CXCR4 signalling are known to play key roles in EPC migration to ischemic tissue, as blockade of these signalling pathways results in a dramatic reduction in EPC homing and consequent impaired neovascularization [120].

### 5.2. EPCs and Diabetes

Hyperglycemia significantly alters endothelial cell biology and leads to a delayed healing response to injuries and vasodegenerative micro- and macrovascular complications. EPC number and function have been shown to be significantly reduced in diabetic patients. Levels of circulating EPCs are reduced by up to 50% in diabetic patients when compared to nondiabetic controls [121]. CD34 expressing progenitors were also found to be reduced in type 2 diabetic patients when compared to healthy controls. Furthermore, these putative EPCs were found to negatively correlate with disease severity [122]. In addition, it has been reported that EPCs derived from diabetic individuals exhibit a diminished vasoreparative potential [123], display an impaired migratory activity, and undergo premature senescence [124, 125]. ECFCs isolated from umbilical cord blood of diabetic patients exhibited reduced colony forming ability and decreased tube forming capacity in matrigel, when compared to ECFCs derived from nondiabetics [125]. It has also been shown that diabetic rats have decreased levels of circulating EPCs as a result of bone marrow neuropathy, and this phenomenon is said to precede the development of diabetic retinopathy [126].

### 5.3. EPC Differentiation and Epigenetics

Heterochromatin protein-1 alpha (HP-1*α*), an epigenetic regulator implicated in gene silencing, has been reported to play an important role in promoting EPC differentiation* in vitro *and* in vivo* [127]. Repressive H3K27me3 and DNA methylation marks were recently reported in eNOS promoters of early EPCs, which were reversed in hypoxic conditions to increase eNOS expression, consequently increasing endothelial recruitment and differentiation [90]. HDAC1, a histone deacetylase enzyme, is also known to play a key role in inhibiting endothelial differentiation and proliferation [128]. The use of epigenetic drugs to reverse these epigenetic marks is being studied recently as a strategy to enhance revascularization [129]. Trichostatin-A is a histone deacetylase inhibitor which has been used to treat ECFCs before transplantation into ischemic tissues in order to increase revascularization efficiency [130].

### 5.4. Why Might Glycemic Memory Be Relevant for EPC Biology?

#### 5.4.1. Progenitor Concept

Although EPCs are considered to be more resistant to oxidative stress than mature endothelial cells, these cells have been shown to be vulnerable in a diabetic environment [131]. Experimental evidence demonstrates that prolonged exposure of EPCs to oxidative stress compromises their reparative potential [132]. EPCs exposed to hyperglycemia for prolonged periods displayed reduced migration and adhesive properties* in vitro* and impaired recruitment to neoendothelium* in vivo.* This phenomenon of EPC dysfunction can be attributed to lowered levels of cathepsin-L activity and upregulation of thrombospondin-1, which were responsible for impaired EPC migration and adhesion, respectively [133, 134].

Nevertheless, when compared to their mature counterparts, progenitor cells, that is, ECFCs/OECs, have been shown to undergo a higher number of population doublings and possess a longer* in vitro* lifespan [135]. Furthermore, umbilical cord blood-derived ECFCs have an enhanced proliferative potential when compared to those obtained from peripheral blood [109]. Mature endothelial cells proliferative capacity is known to be limited; indeed, under high glucose conditions mature endothelial cells readily undergo premature senescence [136]. These data led us to hypothesize that, following exposure to the diabetic milieu, the endogenous, resident pool of reparative endothelial progenitors may become damaged. Indeed, these EPCs have an important vascular homeostatic role and this cellular reserve could serve as potential “carriers” of the endothelial glycemic memory in diabetic patients.

#### 5.4.2. Therapeutic Potential and Modulation of EPCs

EPCs have considerable potential for autologous cell therapy. This involves cell isolation from patients' own blood which are then expanded in culture and given directly back to the same patient. In the context of diabetes, patient-derived EPCs may carry a “memory” of their former hyperglycemic environment which might significantly affect their function. For ECFCs, in particular, this detrimental effect of diabetes has been previously demonstrated. When ECFCs were exposed to high glucose conditions* in vitro* or a diabetic environment* in vivo,* they exhibited diminished function such as reduced colony formation, decreased self-renewal capacity, and tube formation [125]. This was a direct result from exposure to the diabetic milieu. Therefore, there is a need to modulate the function of such “diseased diabetic EPCs/ECFCs” in order to restore normal angiogenic function before their final use as a cytotherapy.

Some reports have started to emerge suggesting various strategies to reverse EPC dysfunction caused by diabetes. Rosiglitazone treatment [137], enhancement of angiogenic stimulus using G-CSF [138], promoting cell deformability, and usage of nitric oxide donor to reverse SDF-1 mediated migration defects [139] are some of the approaches employed to overcome the consequences of EPC dysfunction. However, it must also be taken into consideration that these corrective measures might not be fully successful due to the unfavourable milieu in diabetic tissues [140]. Advanced glycation endproducts cause damage to capillary basement membrane that hinders homing of EPCs. This shortcoming can be overcome by studying EPC interactions with substrates and learning how to modulate these [141]. Modification of the diabetic microenvironment prior to cell delivery is an important area that requires further research.

## 6. Concluding Remarks

Epidemiological and experimental evidence accumulated over the last two decades has proven the existence of a glycemic memory phenomenon triggered by uncontrolled hyperglycemia in the early stages of disease. The consensus view is that an effective way to diminish diabetic vascular complications is early diagnosis and initiation of appropriate glycemic control measures. This will prevent the imprinting of glycemic memory in endothelial (and other) cells and consequently result in a slower progression of vascular complications.

While oxidative stress and AGE accumulation seem to be the key biochemical drivers of glycemic memory, epigenetic changes such as histone modifications and DNA methylation appear to modulate glycemic memory at the molecular level. Furthermore, it seems likely that endogenous endothelial progenitors represent a significant cellular component where all these biochemical and molecular processes take place. Considering their extended lifespan and vascular homeostatic function, it is likely that the glycemic memory affects EPCs and that this contributes to the impaired vasoreparative capacities of diabetic tissues.

There is a pressing need for new therapeutic targets to address the glycemic memory phenomenon. Among many candidates, drugs that modulate epigenetic mechanisms have the potential to “erase” the glycemic memory and consequently delay progression of microvascular complications.

## Figures and Tables

**Table 1:** Summary of clinical trials comparing the effect of intensive and conventional glycemic control on the prognosis of diabetic complications in type 1 and type 2 diabetic patients. ^∗^Related to microalbuminuria and proteinuria levels.

Clinical trial	Type of diabetes	Number of cases	Duration of follow-up (years)	Effect of intensive versus conventional glycemic control on diabetic complications			
				Retinopathy	Neuropathy	Nephropathy	Cardiovascular defects
SDIS	T1D	96	5	+	+	+	NA
DCCT	T1D	1441	6.5	+	+	+	=
DCCT/EDIC	T1D	1375	8	NA	NA	+	+
DCCT/EDIC	T1D	1214	18	+	NA	NA	NA

KPCT	T2D	110	6	+	+	+	NA
STENO2	T2D	160	7.8	+	+	+	+
UKPDS	T2D	3867	10	+	NA	+^∗^	=
VADT	T2D	1791	5.6	=	=	=	=
ADVANCE	T2D	11140	5	=	NA	+	=
ACCORD	T2D	10251	3.5	NA	NA	NA	−

+, benefit; =, no change; −, harm; NA, not assessed.

**Table 2:** Summary of *in vitro* and *in vivo* studies showing epigenetic marks associated with diabetic vascular complications. ^∗^Studies directly related to the endothelial glycemic memory.

Type of epigenetic change	Location	Enzyme	Cell type	Target genes/functions	Reference
HAc and HMe	↑ H3K9Ac, ↑ H3K4me3		T1D monocytes	↑ STAT1, ↑ TNF*α*, and ↑ IL1A	[76]
HAc and HMe	↑ H3K9Ac, ↑ H3K12Ac, ↑ H3K4me2, and ↑ H3K4me3		HUVECs	↑ eNOS	[89]
HMe	↑ H3K27me3		eEPCs	↑ eNOS	[90]
HMe	↑ H3K4me1, ↓ H3K9me2, and ↓ H3K9me3	Set7, SUV39h1, and LSD-1	HG HMECs	↑ NF*κ*B P65	[80]
HMe	↑ H3K4me1	Set7	HG HMECs	↑ ICAM1, ↑ IL8, and ↓ HMOX1	[81]
HMe	↓ H3K4me1, ↓ H3K4me2	LSD-1	HG rat retinal ECs	↓ Sod2	[77]
HAc	↓ H3 acetylation	↓ HAT ↑ HDAC1, HDAC2, and HDAC8	HG rat retinal ECs^∗^		[79]
HAc	↑ H3 acetylation	HAT cofactor p300	HG HUVECs^∗^	↑ ET-1, ↑ FN, ↑ VEGF, ↑ NF*κ*B, ↑ AP-1, ↑ MEF2, and ↑ GATA	[83]
HMe	↑ H3K4me	Set7/9	Monocytes	↑ NF*κ*B, ↑ TNF*α*, ↑ RAGE, and ↑ JMJD3; ↑ monocyte adhesion to ECs	[82]

DNAme	↑ Promoter of UNC13B		T1D whole blood cells	↓ UNC13B; ↑ chronic kidney disease	[91]
DNAme	↑ Promoter of POLG1		HG rat retinal Ecs^∗^	↓ POLG1; ↑ glomerular apoptosis, ↑ d-loop damage in mt DNA	[93]
DNAme	↑ Global hypomethylation		Diabetic foot ulcer fibroblasts^∗^	↓ Wound healing process	[92]

HAc, histone acetylation; HMe, histone methylation; DNAme, DNA methylation; HG, hyperglycemic.
